# Creation of Wood‐Based Hierarchical Superstructures via In Situ Growth of ZIF‐8 for Enhancing Mechanical Strength and Electromagnetic Shielding Performance

**DOI:** 10.1002/advs.202400074

**Published:** 2024-02-21

**Authors:** Haoran Ye, Ying Wu, Xin Jin, Jiamin Wu, Lu Gan, Jianzhang Li, Liping Cai, Chuangwei Liu, Changlei Xia

**Affiliations:** ^1^ Jiangsu Co‐Innovation Center of Efficient Processing and Utilization of Forest Resources International Innovation Center for Forest Chemicals and Materials College of Materials Science and Engineering Nanjing Forestry University Nanjing Jiangsu 210037 China; ^2^ Key Lab for Anisotropy and Texture of Materials School of Materials Science and Engineering Northeastern University Shenyang 110819 China

**Keywords:** antimicrobial properties, electromagnetic shielding, high strength, metal–organic framework, self‐bonding

## Abstract

Given the escalating prevalence of electromagnetic pollution, there is an urgent need for the development of high‐performance electromagnetic interference (EMI) shielding materials. Herein, wood‐based electromagnetic shielding materials have gained significant popularity due to their exceptional performance as building materials. In this study, a novel wood‐based composite with electromagnetic shielding properties is developed. Through the in situ growth of zeolitic imidazolate framework‐8 (ZIF‐8) crystals on wood fibers, coupled with uniform integration of carbon nanotubes (CNTs), a multifunctional composite named ZIF‐8/Poplar‐CNT composite is synthesized via a one‐step thermoforming process. The incorporation of CNTs endows the composites with excellent EMI shielding effectiveness (EMI SE). Among these elements, despite ZIF‐8 crystals not possessing intrinsic electromagnetic shielding functionality, their distinctive dodecahedral structure proves adept at scattering and reflecting electromagnetic waves within the composites, further improving the electromagnetic shielding effect. Hence, the ZIF‐8/Poplar‐CNT composite (56.95 dB) has ≈10 dB higher EMI SE compared to that of the composites without ZIF‐8 crystals. Meanwhile, ZIF‐8 crystals endow the materials with excellent tensile strength (54.84 MPa, enhanced by 4 times). Moreover, the introduction of Zn^2+^ provides superior antibacterial properties. The potential applications of ZIF‐8/Poplar‐CNT composites extend to diverse areas such as building decoration, electronic products, and medical equipment.

## Graphical abstract

This study reports a facile fabrication process of self‐bonding MOF/wood‐based composites with excellent mechanical properties, efficient electromagnetic shielding, and antibacterial properties. The composites can be used not only for interior decoration but also for exterior decoration and public places to reduce electromagnetic radiation and bacterial reproduction.

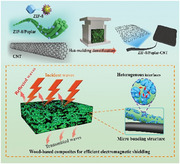

## 1 Introduction

In tandem with the rapid progress of modern science and technology, the electronics industry has experienced significant advancements.^[^ ^1^ ^]^ The extensive utilization of various electronic devices has greatly facilitated people's lives, providing substantial convenience.^[^ ^2^ ^]^ However, the ensuing electromagnetic pollution problem has become increasingly severe.^[^ ^3^ ^]^ Especially for high‐frequency equipment, the output energy is immense during the working period, and the formed electromagnetic radiation is also powerful, which would cause severe interference to the surrounding electronic equipment, instruments, and information signals, so that it cannot work typically and cause adverse consequences.^[^ ^4^ ^]^ Meanwhile, if it is strongly impacted by electromagnetic radiation, there is a risk of stolen military secrets, and the equipment information system would also be temporarily disabled or permanently damaged.^[^ ^5^ ^]^ On the other hand, long‐term exposure to electromagnetic radiation can cause irreparable damage to the human body, leading to DNA damage and serious diseases such as cancer and leukemia.^[^ ^6^ ^]^ Furthermore, excessive electromagnetic radiation can also affect the natural environment on which humans depend for survival.^[^ ^7^ ^]^ Hence, to effectively prevent and control electromagnetic pollution, preparing high‐performance EMI shielding materials has gradually become a new research hotspot.^[^ ^8^ ^]^

Traditional EMI shielding materials are usually prepared based on metal materials, and the shielding effectiveness (SE) is achieved by weakening the interference of high‐frequency magnetic fields.^[^ ^9^ ^]^ However, it should be noted that while metal‐based electromagnetic shielding materials may offer resistance to electromagnetic waves, there remains the possibility of some frequency ranges allowing for the penetration of such waves.^[^ ^10^ ^]^ This can result in the occurrence of secondary electromagnetic pollution during the course of usage.^[^ ^11^ ^]^ Moreover, they have inherent drawbacks, such as high cost, high density, and complex processing, which cannot meet the production needs of portable equipment.^[^ ^12^ ^]^ Compared with them, polymer electromagnetic shielding materials have the benefits of adjustable conductivity, lightweight, and excellent processing performance.^[^ ^13^ ^]^ However, such electromagnetic shielding materials still have some drawbacks, such as poor mechanical strength and easy deformation.^[^ ^14^ ^]^ At present, carbon‐based electromagnetic shielding materials are considered as promising electromagnetic shielding materials owing to their excellent strength and electrical conductivity.^[^ ^15^ ^]^ With the continuous exploration of renewable resources, biomass functional materials based on biomass have developed rapidly.^[^ ^16^ ^]^ As the most widely used renewable resource, wood has the advantages of lightweight, environmental protection, low cost, and easy processing.^[^ ^17^ ^]^ Hence, combining pretreated wood with carbon‐based materials can prepare high‐performance wood‐based electromagnetic shielding materials. CNTs are a class of carbon‐based materials that exhibit exceptional thermal and electrical conductivity. These nanoscale tubular structures, composed of carbon atoms, are frequently employed in the fabrication of wood‐based electromagnetic shielding composites. Nonetheless, the current preparation process presents several deficiencies, such as intricate procedures, inadequate bonding performance, and insufficient mechanical properties. Accordingly, there is a pressing need to develop novel preparation techniques that better address these issues.^[^ ^18^ ^]^

Metal–organic framework (MOF) is a kind of coordination polymers formed by the interconnection of metal ions and organic ligands.^[^ ^19^ ^]^ Owing to their advantages of extremely high specific surface area, large porosity, structural diversity, and versatility.^[^ ^20^ ^]^ MOFs are often combined with other materials to prepare multifunctional materials with excellent mechanical properties.^[^ ^21^ ^]^ Due to the abundant hydroxyl groups in wood, chemical pretreatments can be used to provide adjustable chemically active sites.^[^ ^22^ ^]^ It is very suitable for in situ growth of MOFs. In situ growth of MOFs in wood can improve the strength of wood to a certain extent.^[^ ^23^ ^]^ Currently, the integration of Metal–Organic Frameworks (MOFs) with wood fibers has been found to produce sustainable composite materials that are highly suitable for various construction applications. Hence, it is of excellent research meaning to combine MOF/wood‐based composites with carbon‐based materials to prepare materials with efficient electromagnetic shielding properties using a simple preparation method.^[^ ^24^ ^]^

**Figure 1:**
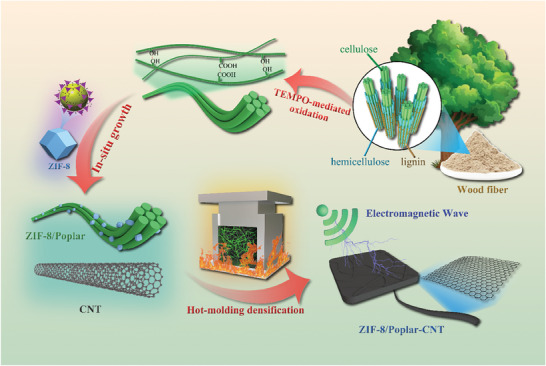
Schematic diagram of the fabrication process of ZIF‐8/Poplar‐CNT composite with efficient EMI SE.

In this study, ZIF‐8 crystals are in situ grown on TEMPO‐oxidized wood powder (TEMPO: 2, 2, 6, 6‐tetramethylpiperidinyl‐1‐oxide) to obtain ZIF‐8/Poplar composites. Then, the multifunctional ZIF‐8/Poplar‐CNT composite with excellent mechanical properties is prepared by uniformly mixing with CNTs using a one‐step thermoforming method (**Figure** **1**). The in situ growth of ZIF‐8 crystals endows material with a stable chemical structure, and self‐bonding occurs between wood fibers under the action of thermal molding, which provides mechanical strength support for ZIF‐8/Poplar‐CNT composites. The intervention of CNTs endows the composites with excellent electromagnetic interference shielding effectiveness (EMI SE). Therefore, the unique dodecahedral structure of ZIF‐8 crystals can effectively scatter and reflect electromagnetic waves in the composites, thereby reducing the propagation and penetration ability of electromagnetic waves and providing more efficient EMI shielding performance for the ZIF‐8/Poplar‐CNT composite. The antibacterial effect of zinc ions (Zn^2+^) on various bacteria makes the ZIF‐8/Poplar‐CNT composite have good antibacterial properties. Furthermore, other test characterizations show that the material has excellent thermal conductivity and flame retardancy. In summary, ZIF‐8/Poplar‐CNT composites can be applied in interior decoration and public places to reduce electromagnetic radiation and bacterial proliferation, providing a clean and hygienic environment. It can also be applied as the shell or substrate for electronic and medical equipment to reduce the growth of bacteria and electromagnetic interference. Moreover, the ZIF‐8/Poplar‐CNT composite uses renewable biomass as its raw material, and the preparation process is characterized by its simplicity, aligning the research with principles of sustainable development.

## 2 Results and Discussion

### 2.1 Characterization of ZIF/Poplar Composite

**Figure 2:**
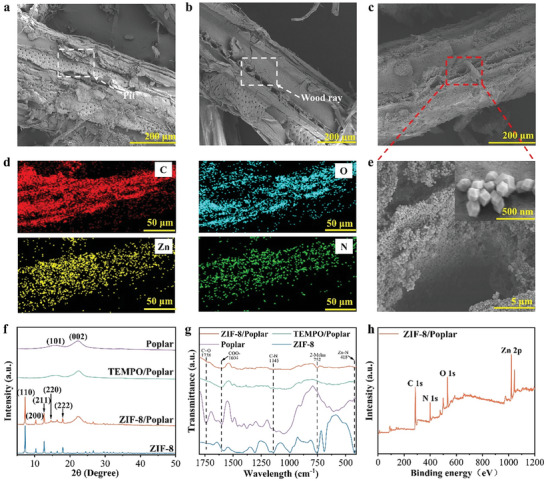
SEM images of the a) poplar, b) TEMPO/Poplar composite, and c,e) ZIF‐8/Poplar composite. d) The EDS element distribution diagram of ZIF‐8/Poplar composite corresponding to Figure c. f) XRD patterns and g) FTIR spectra of Poplar, TEMPO/Poplar composite, and ZIF‐8/Poplar composite. h) XPS total spectra of ZIF‐8/Poplar composite.

Initially, natural wood fibers undergo pretreatment with an alkali solution. This process involves the partial removal of lignin and hemicellulose, thereby exposing the internal cellulose structure.^[^ ^25^ ^]^ After TEMPO oxidation, the hydroxyl groups (−OH) on the surface of cellulose are converted into carboxyl groups (−COOH).^[^ ^26^ ^]^ This step ensures the ion exchange between Zn^2+^ and −COOH, thereby providing active chemical sites for the subsequent in situ growth of ZIF‐8 crystals. It can be observed from the scanning electron microscopy (SEM) images that the wood fiber structure is distinct, with typical pit characteristics (**Figure** **2**a), and its internal structure is not damaged after the TEMPO oxidation (Figure 2b).^[^ ^27^ ^]^ On the surface of the ZIF/Poplar composite, a layer of dense dodecahedral nanocrystals (ZIF‐8 crystals) can be observed to grow uniformly on the cell walls of wood fibers (Figure 2c,e). Energy dispersive spectra (EDS) element distribution diagram of the ZIF/Poplar composite detects C, N, O, and Zn elements (Zn only originates from ZIF‐8 crystals) (Figure 2d).^[^ ^28^ ^]^ The in situ‐grown ZIF‐8 crystals have many micropores and mesopores, which can increase the specific surface area of the ZIF‐8/Poplar composite (429.46 m^2^ g^−1^) (Figure S1, Supporting Information).^[^ ^29^ ^]^ As shown in Figure 2f, the X‐ray diffraction (XRD) pattern of ZIF/Poplar composite is consistent with those of natural wood and TEMPO/Poplar composite, so its crystal structure remains unchanged. Meanwhile, the (110), (200), (211), (220), and (222) planes show diffraction peaks consistent with ZIF‐8 crystals.^[^ ^30^ ^]^ This means the successful in situ growth of ZIF‐8 crystals within the ZIF/Poplar composite. The surface functional group changes of wood fibers and the ZIF/Poplar composite before and after TEMPO oxidation are further analyzed by the Fourier transform infrared spectrometer (FT‐IR) (Figure 2g). Compared with natural wood fibers, the carbonyl (−C═O) intensity of TEMPO‐oxidized wood fibers is significantly decreased at 1738 cm^−1^, while the −COOH intensity at 1604 cm^−1^ is increased, which demonstrates the apparent removal of hemicellulose and the conversion of −OH in cellulose to −COOH. The characteristic peaks of the ZIF/Poplar composite at Zn–N (419 cm^−1^), 2‐MeIm (752 cm^−1^), and C–N (1145 cm^−1^) are consistent with those of ZIF‐8 crystals.^[^ ^31^ ^]^ Moreover, the X‐ray photoelectron spectroscopy (XPS) total spectrum proves the existence of C, N, O, and Zn elements in the ZIF‐8/Poplar composite, and their atomic contents are 52.97%, 14.54%, 15.52%, and 16.97%, respectively (Figure 2h).^[^ ^32^ ^]^ In conclusion, ZIF‐8 crystals are successfully grown in situ on the surface of wood fibers of the ZIF/Poplar composite.

### 2.2 Morphology and Physical Properties of ZIF‐8/Poplar‐CNT Composite

**Figure 3:**
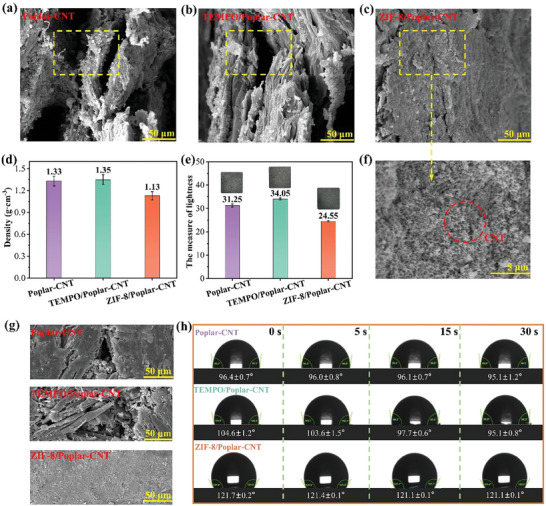
SEM images of the a) Poplar‐CNT composite, b) TEMPO/Poplar‐CNT composite, and c,f) ZIF‐8/Poplar‐CNT composite (cross‐section). d) Density, e) Lightness comparison, g) SEM images (surface), and h) Contact angles of Poplar‐CNT composite, TEMPO/Poplar‐CNT composite, and ZIF‐8/Poplar‐CNT composite.

**Figure** **3**a–c schematically indicates the ZIF‐8/Poplar‐CNT composite has a dense internal structure with almost no pores. This is because the loading of ZIF‐8 crystals can form consecutive covering layers on the surface of wood fibers, which enhances the interfacial bonding between fibers and prevents their interlayer separation.^[^ ^33^ ^]^ Furthermore, the one‐step thermoforming method provides hot‐pressing conditions for preparing the ZIF‐8/Poplar‐CNT composite, forming a dense structure.^[^ ^34^ ^]^ Figure 3f shows the uniform dispersion of CNTs in the ZIF‐8/Poplar CNT composite. Hence, the ZIF‐8/Poplar‐CNT composite has a lower density (1.13 g·cm^−3^) (Figure 3d).^[^ ^35^ ^]^ Owing to the uniform dispersion of CNTs, the lightness (24.55) of the ZIF‐8/Poplar‐CNT composite is observed to decrease significantly (Figure 3e). As shown in Figure 3g, the surface of the ZIF‐8/Poplar‐CNT composite is dense and smooth without obvious cracks, which makes it difficult for moisture to enter from the surface. Moreover, when ZIF‐8 crystals are grown in situ on wood flour and subsequently consolidated into sheets through hot‐pressing, a robust composite structure is formed, imparting additional mechanical stability and protection to the ZIF‐8/Poplar‐CNT composite. The resulting stable architecture effectively retards the deliquescence of ZIF‐8 crystals in the presence of humidity and air by creating a protective environment. This feature maintains the contact angles of the ZIF‐8/Poplar‐CNT composite at ≈120° within 30s, demonstrating excellent hydrophobic properties (Figure 3h).^[^ ^36^ ^]^ In summary, in situ, growth of ZIF‐8 crystals on wood fibers can promote the combination with CNTs and improve the dimensional stability of the ZIF‐8/Poplar‐CNT composite.

### 2.3 Mechanical Properties of ZIF‐8/Poplar‐CNT Composite

**Figure 4:**
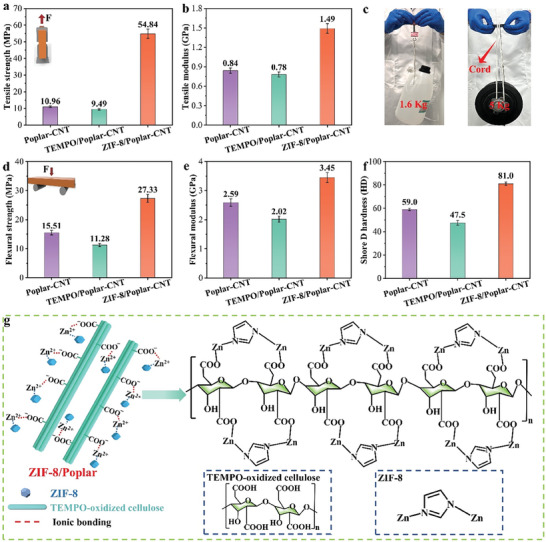
a) Tensile strength, b) Tensile modulus, d) Flexural strength, e) Flexural modulus, and f) Shore D hardness of Poplar‐CNT composite, TEMPO/Poplar‐CNT composite and ZIF‐8/Poplar‐CNT composite. c) The load‐bearing photograph of ZIF‐8/Poplar‐CNT composite. g) Schematic diagram of the cross‐linking network of ZIF‐8/Poplar composite.

Excellent mechanical strength is essential to support any material in practical applications. The mechanical strength of ZIF‐8/Poplar‐CNT composite has been significantly improved due to the loading of ZIF‐8 crystals. Specifically, the tensile strength (54.84 MPa) and flexural strength (27.33 MPa) of the ZIF‐8/Poplar‐CNT composite are much higher than those of the Poplar‐CNT composite and TEMOP/Poplar‐CNT composite (**Figure** **4**a,d). Meanwhile, the tensile and flexural modulus of the ZIF‐8/Poplar‐CNT composite are also consistent with the above changing rules (Figure 4b,e).^[^ ^37^ ^]^ The corresponding stress–strain curves are characterized in Figure S2 (Supporting Information). From Figure 4c, it can be seen intuitively that the ZIF‐8/Poplar‐CNT composite can withstand a 1.6 kg bucket without breaking and can serve as a beam to support a 5 kg dumbbell without being broken.^[^ ^38^ ^]^ Furthermore, the Shore D hardness values of Poplar/CNT composite and TEMPOP/Poplar CNT composite are 59 and 47.5, respectively, whereas that of ZIF‐8/Poplar CNT composite are as high as ≈81, exceeding the typical hardness values of polymers and hardwoods (Figure 4f). This is owing to the ion exchange between the negative charges on −COOH in the TEMPO‐oxidized cellulose and Zn^2+^, resulting in a more stable chemical structure (Figure 4g). Meanwhile, ZIF‐8 crystals and CNTs are physically adsorbed due to the interaction of van der Waals force, so that the CNTs are fixed on the wood fibers. Moreover, the hydroxyl groups on wood fibers can provide active sites for the functional groups on the surface of CNTs. They chemically bond or coordinately bond with the metal ions or organic ligands of ZIF‐8 crystals, enhancing their bonding strength.^[^ ^39^ ^]^ This stable structure enhances the self‐bonding of the materials, and the ZIF‐8/Poplar‐CNT composite has excellent mechanical properties with the help of hot‐pressing, providing a solid mechanical foundation for its practical applications.

### 2.4 Electrical Conductivity and Electromagnetic Shielding Performance of ZIF‐8/Poplar‐CNT Composite

**Figure 5:**
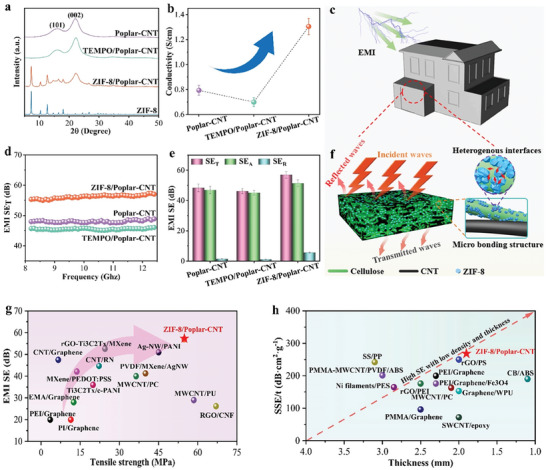
a) XRD patterns, b) Electrical conductivity, d) EMI SE_T_, e) EMI SE (SE_T_, SE_A_, SE_R_) of Poplar‐CNT composite, TEMPO/Poplar‐CNT composite, and ZIF‐8/Poplar‐CNT composite. c) Schematic diagram of a high EMI shielding building constructed by ZIF‐8/Poplar‐CNT composite. f) EMI shielding mechanism diagram of ZIF‐8/Poplar‐CNT composite. g) The mechanical strength and EMI SE of ZIF‐8/Poplar‐CNT composite compared with reported EMI shielding materials (Table S1, Supporting Information). h) Comparison of SSE/t versus thickness (Table S2, Supporting Information).

Conductivity is an essential basis for measuring the EMI SE of materials.^[^ ^40^ ^]^ **Figure** **5**a shows the ZIF‐8/Poplar‐CNT composite has a characteristic peak similar to that of ZIF‐8 crystals. This indicates that the hot‐pressing does not alter the crystal type of the material. Upon comparing the position and shape of the XRD peaks with Figure 2f, it is evident that the chemical properties of the composite remain unaffected by the intervention of CNTs. The result suggests that the incorporation of CNTs does not alter the underlying chemical structure of the composite, thereby preserving its original properties. Meanwhile, the in situ growth of ZIF‐8 crystals is capable of promoting the amalgamation of wood fibers and CNTs, enhancing the propagation of electromagnetic waves in the ZIF‐8/Poplar‐CNT composite. Hence, the ZIF‐8/Poplar‐CNT composite has greater conductivity (Figure 5b).^[^ ^41^ ^]^ Specifically, when the ZIF‐8/Poplar‐CNT composite is connected to the channel, the LED light immediately emits a bright green light (Figure S3a, Supporting Information), which further proves its excellent electrical conductivity. Natural wood with excellent workability is often classified as an insulators. After mixing CNTs, the combination of the two would be terrible due to the difference in surface properties. However, the intervention of ZIF‐8 crystals enhances the interface combination of the two, which is conducive to the rapid transfer of electrons.

Figure 5d characterizes the EMI SE of the composites in the frequency range of 8.2–12.4 GHz (X‐band). Obviously, the total shielding effectiveness (SE_T_) of the composites increases from 46.21 to 56.95 dB after in situ growth of ZIF‐8 crystals. It well exceeds the EMI SE (20 dB) required in commercial applications. The reflection efficiency (SE_R_) and absorption efficiency (SE_A_) values, derived using the measured s parameters, are presented in Figure 5e. Obviously, the ZIF‐8/Poplar‐CNT composites display the highest SE_R_ and SE_A_ values. On this basis, the average values of reflection coefficient (R), absorption coefficient (A), and transmission coefficient (T) are employed to analyze the ZIF‐8/Poplar‐CNT composite. Notably, the R‐value of ZIF‐8/Poplar‐CNT composite is greater than the A value, which proves that it mainly reflects electromagnetic waves (Figure S3b, Supporting Information). Hence, the reflection consumption is the primary electromagnetic shielding mechanism of ZIF‐8/Poplar‐CNT composites.^[^ ^42^ ^]^ This characteristic enables ZIF‐8/Poplar‐CNT composites to be applied as a green and sustainable EMI shielding material. As shown in Figure 5g, compared with common EMI shielding materials, the ZIF‐8/Poplar‐CNT composite has a significant advantage in tensile strength when EMI SE is similar. Furthermore, the specific EMI SE (SSE/t = SE/density/thickness) values are also critical indicators to measure EMI shielding performance. The recent advancements in EMI shielding materials have propelled the specific EMI SE beyond 105 dB·cm^2^·g^−1^. The ZIF‐8/Poplar‐CNT composite boasts an impressive SSE/t value of 263.86 dB·cm^2^·g^−1^ (Figure 5h), outperforming other common EMI shielding materials of its kind.^[^ ^43^ ^]^ In summary, the excellent mechanical strength and electromagnetic shielding properties of the ZIF‐8/Poplar‐CNT composite enable it to be applied as a building material to ensure the safety of internal electronic equipment (Figure 5c).^[^ ^44^ ^]^ It can also serve as a protective shell for medical equipment to prevent external electromagnetic interference from affecting its working effect. Moreover, the lower density would significantly expand the practical applications of the ZIF‐8/Poplar‐CNT composite in aerospace, military, and portable electronics.

The electromagnetic shielding mechanism diagram of the ZIF‐8/Poplar‐CNT composite is presented in Figure 5f. When the electromagnetic waves are incident on the ZIF‐8/Poplar‐CNT composite, most of them are reflected by it. This is owing to the superior electrical conductivity of CNTs. After in situ growth of ZIF‐8 crystals, wood fibers can promote the combination with CNTs, which enhances the conductivity of electromagnetic waves in ZIF‐8/Polar CNT composites.^[^ ^45^ ^]^ Nevertheless, the electric field in electromagnetic waves can induce the movement of free electrons in the ZIF‐8/Poplar‐CNT composite, thereby generating an electric current. The formation of this current will hinder the propagation of electromagnetic waves in the ZIF‐8/Poplar‐CNT composite, which means that the attenuation of electromagnetic waves increases, thereby achieving electromagnetic shielding effects.^[^ ^46^ ^]^ Meanwhile, the unique dodecahedral structure of the ZIF‐8 crystal can effectively scatter and reflect electromagnetic waves inside the composites, thereby reducing the propagation and penetration capabilities of electromagnetic waves and further improving the electromagnetic shielding effect.^[^ ^47^ ^]^ Furthermore, the ZIF‐8/Poplar‐CNT composite after hot‐pressing has a dense interfacial bonding, which can enhance the reflection and scattering of electromagnetic waves, thereby effectively weakening the propagation ability of electromagnetic waves.

### 2.5 Thermal Properties, Antibacterial Performance, and Life‐Cycle Assessment of ZIF‐8/Poplar‐CNT Composite

**Figure 6:**
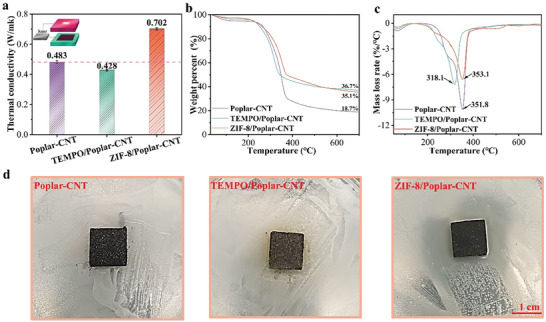
a) Thermal conductivity comparison, b) TG curves, c) DTG curves, and d) Antibacterial properties of Poplar‐CNT composite, TEMPO/Poplar‐CNT composite, and ZIF‐8/Poplar‐CNT composite.

Usually, general electromagnetic shielding equipment is prone to heat and damage the equipment due to long‐term radiation of electromagnetic waves, so excellent heat dissipation performance is indispensable. **Figure** **6**a characterizes that the thermal conductivity of the ZIF‐8/Poplar‐CNT composite (0.702 W mk^−1^) is higher than that of the Poplar‐CNT composite (0.483 W mk^−1^) and TEMPO/Poplar‐CNT composite (0.428 W mk^−1^).^[^ ^48^ ^]^ The microstructure of ZIF‐8 crystals plays a significant role in the increased effectiveness of heat transfer. This is due to its ability to enhance the contact surface area with the wood floor. Furthermore, ZIF‐8 forms a robust interface bond with wood powder through in situ growth. This bond results in a reduction in interface thermal resistance and the promotion of heat conduction. The excellent thermal conductivity helps ZIF‐8/Poplar‐CNT composite to disperse and diffuse heat, reducing the formation of hot spots. Thermogravimetric analysis (TGA) curves are characterized the thermal stability of composites (Figure 6b). The residual mass of the Poplar‐CNT composite at 700 °C is 18.7%. Significantly, the residual mass of the ZIF‐8/Poplar‐CNT composite is 35.1%. This indicates that the loading of ZIF‐8 crystals can inhibit the thermal decomposition of wood to a certain extent. Moreover, DTG curves reveal that the maximum weight loss temperature of the ZIF‐8/poplar CNT composite was 353.1 °C, which was higher than that of the Poplar‐CNT composite (318.1 °C) and TEMPO/Poplar‐CNT composite (351.8 °C). Meanwhile, the maximum degradation rate of the ZIF‐8/Poplar‐CNT composite is much slower than that of the Poplar‐CNT composite and TEMPO/Poplar‐CNT composite (Figure 6c). The alcohol lamp burning experiment further explores the combustion behavior of the ZIF‐8/Poplar‐CNT composite (Figure S4, Supporting Information). The results indicate that the ZIF‐8/Poplar‐CNT composite has good flame retardancy. The main reason is that a protective layer is formed by the one‐step thermoforming after the ZIF‐8 crystals are loaded, resulting in less heat release. Additionally, in conditions of elevated temperature, ZIF‐8 crystals undergo decomposition, resulting in the creation of a carbon residue. This residue, in turn, forms a protective layer on the surface of the wood powder, exhibiting an ability to resist flames and oxygen. Consequently, it impedes the propagation of flames and enhances the flame retardation properties of the material. Antibacterial tests show *Escherichia coli* thrives on the Poplar‐CNT composite and TEMPO/Poplar‐CNT composite, while there is a clear antibacterial zone around the ZIF‐8/Poplar CNT composite (Figure 6d).^[^ ^49^ ^]^ This reveals that the ZIF‐8/Poplar CNT composite has excellent antibacterial properties. This is because the loading of ZIF‐8 crystals introduces zinc ions with antibacterial properties into composites. Hence, the ZIF‐8/Poplar‐CNT composite can be used in medical equipment and instruments to reduce the breeding of bacteria and pathogens to improve the hygiene level of medical environments. It can also be used as a countertop in the catering industry to improve food hygiene and safety.

Life‐cycle assessment (LCA) was conducted on four materials, that is ZIF‐8/Poplar‐CNT composite, copper, steel, and aluminum, using the evaluation criteria of Building for Environmental and Economic Sustainability (BEES) and eco‐indicators (Pt). The life‐cycle inventory (LCI) indicates that the environmental impact index of the ZIF‐8/Poplar‐CNT composite is much lower than that of common electromagnetic shielding metal materials (Figure S5 and Table S3, Supporting Information).^[^ ^50^ ^]^ Moreover, the total environmental impact values of the four materials calculated using eco‐indicators were 1011.65, 268.71, 17.45, and 3.65 Pt, respectively (Figure S3c, Supporting Information). Obviously, compared with each metal material, the total environmental impact values of ZIF‐8/Poplar‐CNT composite are reduced by 276.16, 72.61, and 3.78 times, respectively.^[^ ^51^ ^]^ Hence, the application of the ZIF‐8/Poplar‐CNT composite can better reduce the environmental pollution caused by metal materials.

## 3 Conclusion

This study presents a facile fabrication process for self‐bonding MOF/wood‐based composites, showcasing remarkable mechanical attributes, proficient electromagnetic shielding capabilities, and antibacterial properties. As a result, the ZIF‐8/Poplar‐CNT composite exhibits outstanding tensile strength (54.84 MPa) and flexural strength using the one‐step thermoforming method, ensuring the physical basis of composites in practical applications. It is beneficial to promote its interfacial binding with CNTs by in situ growth of ZIF‐8 crystals on wood fibers. The intervention of CNTs endows the composites with excellent electromagnetic interference shielding effectiveness (EMI SE). Therefore, although ZIF‐8 crystals do not have electromagnetic shielding functionality, their unique dodecahedral structure can effectively scatter and reflect electromagnetic waves inside the composites. This makes the ZIF‐8/Poplar‐CNT composite have EMI SE as high as 56.95 dB (X‐band), which is ≈10 dB greater than that of the composites without ZIF‐8 crystals. Furthermore, Zn^2+^ endows ZIF‐8/Poplar‐CNT composite with remarkable antibacterial properties. It also possesses excellent thermal conductivity and thermal stability. The conducted LCA evaluation reveals that this straightforward preparation process holds promise for wood to replace conventional non‐renewable EMI shielding materials. This transition marks a crucial area of focus in the realm of electromagnetic shielding research. Hence, it is promising to apply ZIF‐8/Poplar‐CNT composites for interior decoration and public places to diminish electromagnetic radiation and bacterial proliferation. It can also function as a protective shell or substrate for electronic and medical equipment, effectively curtailing the propagation of electromagnetic waves and the proliferation of bacteria.

## 4 Experimental Section

### Materials and Chemicals

The poplar (*Populus tremula*) was purchased from the Dongguan Engineering Wood Factory in China and then crushed into 60–80 mesh powder. Zinc nitrate hexahydrate (Zn(NO_3_)_2_·6H_2_O, C_9_H_18_NO, 98%), 2‐Methylimidazole (2‐MeIm, C_6_H_6_N_2_, 97%), and methyl alcohol (≥ 99.5%) were obtained from Wuhan Kamik Co., Ltd., China. The CNTs (tube diameter: 3–5 nm, tube length: 15–30 µm, specific surface area: 250–270 m^2^ g^−1^) were selected from Shenzhen Suiheng Technology Co., Ltd., China. Ethanol (C_2_H_6_O, 99.7%), sodium hydroxide (NaOH, 96%), anhydrous sodium sulfite (Na_2_SO_3_, 98%), sodium hypochlorite (NaClO, 7.5%), sodium bromide (NaBr, 99%), and TEMPO were supplied by Shanghai Macklin Biochemical Co., Ltd., China. The chemicals were applied directly in experiments. Deionized (DI) water was collected from the water purification system.

### Synthesis of ZIF‐8/Poplar Composite

First, 60–80 mesh poplar powders were soaked in the mixed aqueous solution of 0.4 m Na_2_SO_3_ and 2.5 m NaOH, stirred, and heated at 96 °C for 4 h to remove part of lignin and hemicellulose. Then they were rinsed to neutral with deionized water and freeze‐dried to obtain pretreated poplar powder. Second, the TEMPO oxidation reaction was performed according to the procedure described in the report.^[^ ^27^ ^]^ The pretreated poplar powder (30 g), TEMPO (0.48 g), and NaBr (3 g) were sequentially added into 500 mL of deionized water in sequence, stirred thoroughly, and then sonicated for 30 min to achieve uniform dispersion. Subsequently, 100 mL NaClO solution was added into the reaction system after ultrasound treatment. The pH value was controlled ≈10.3 by continually dropping NaOH solution (0.5 mol L^−1^). After 1 h, the oxidation reaction was quenched with ethanol, rinsed with deionized water until neutral, and then freeze‐dried to obtain TEMPO‐oxidized poplar powder, which was called TEMPO/Poplar. Finally, the TEMPO/Poplar (6 g) was vacuum stirred for 3 h in a Zn(NO_3_)_2_ solution, which was obtained by dissolving Zn(NO_3_)_2_·6H_2_O (9.6 g) in methanol (80 g) and DI water (12 g), to ensure the full coordination of Zn^2+^ and carboxyl. Next, the 2‐MeIm solution prepared by dissolving 2‐MeIm (18 g) in methanol (80 g) and DI water (12 g) was added to the above system. After uniform stirring for 20 h, the resulting material was washed with 40 mL of methanol for 30 min and freeze‐dried to obtain ZIF‐8/Poplar composite.

### Fabrication of ZIF‐8/Poplar‐CNT Composite

6 g ZIF‐8/Poplar composite and 0.48 g CNT (8% of ZIF‐8/Poplar composite) were thoroughly mixed using a high‐speed rotary mixer (ZG‐L74A, Chigo, China), and evenly spread in a mold (length × width = 50 mm × 50 mm). After hot‐molding at 180 °C and 30 MPa for 1 h in the hot‐pressing machine (Guangdong Zhenggong Electromechanical Equipment Technology Co., Ltd., China.), the resulting materials were cooled down to 25 °C, and the final products that was called ZIF‐8/Poplar‐CNT composite was accomplished. Furthermore, poplar and TEMPO/Poplar composite were compounded with CNT according to the above preparation process to make two products named Poplar‐CNT composite and TEMPO/Poplar‐CNT composite.

### Physical Characterizations and Antibacterial Performance

Each sample was analyzed in parallel according to the standard of GB/T 17657‐2022 in mechanical testing. The AGS‐X universal mechanical testing machine (Shimadzu, Japan) was applied to investigate the composites. Specifically, the test was performed on samples with a size of 50 mm × 7 mm × 2 mm at room temperature. The crosshead speed of the tensile test was 1.6 mm min^−1^, and the upper and lower spans were 16.5 mm. For bending testing, the three‐point bending equipment was applied with a span of 25 mm and a crosshead speed of 20 mm min^−1^. The DSA100S contact angle measuring instrument (KRUSS) was applied to perform the contact‐angle of composites under the condition of dropping 1.5 µL of DI water each time. Meanwhile, the contact‐angles were recorded within 30 s. The lightness of the composites was measured using an SR60 colorimeter purchased from Guangdong Sanfeng Precision Measuring Instrument Co., Ltd., China. The BET analysis was run on an automated surface area and porosity analyzer (PS4‐1501, Beijing Best Instrument Technology Co., Ltd, China) to determine the specific surface area of composites. In the thermal conductivity test, the prepared composites were characterized by a thermal conductivity tester (DRPL‐2B, Xiangtan Instrument Co., Ltd., China). TGA was analyzed using a TG209F3 thermogravimetric analyzer (NETZSCH, Germany) at a heating rate of 10 °C min^−1^ from 28 to 780 °C in a nitrogen atmosphere. The flame retardancy of the composites was tested by burning for 8s using an alcohol lamp. The electrical conductivity of the composites was characterized using the RTS‐9 dual electric four‐probe tester. The EMI SE of composites was tested with a vector network analyzer (E5063A, Keysight Technology Co., Ltd., China). The test method was the waveguide method, the frequency range was 8.2–12.4 GHz (X‐band), the output power was −15 dBm, and the measured samples’ size was 50 × 50 × 2 mm^3^ (length × width × thickness). The morphology of the composites was characterized using a Regulus 8100 SEM (Hitachi High‐Tech Corporation, China). EDS of the composites were tested with the same instrument. The antibacterial properties of the samples were assessed according to the distribution range of Escherichia coli using the experimental method of Ye et al.

### Chemical Characterization

The functional groups of the composites were characterized by the FT‐IR spectra (Nicolet IS50, Thermo Fisher Technologies, USA) using potassium bromide (KBr) tablet pressing method in the wavelength range of 525–1800 cm^−1^. The internal structure of the composites was characterized by XRD (Beijing Puwei General Instrument Co., Ltd., China) within the scanning range of 5°–50° (2*θ*). The composites were tested by XPS (Shimadsu Enterprise Management Co., Ltd., China), and the elemental composition and molecular structure of the composites were compared by peak position and peak shape.

### Life‐Cycle Assessment

LCA of ZIF‐8/Poplar‐CNT composites, copper, steel, and aluminum was carried out using SimaPro (Version 9.0.0.48) software.^[^ ^51^ ^]^ Meanwhile, the Building for BEES evaluation standard and ecological indicators were used to calculate the LCI, and the impact category was divided into 13 impacts (Table S3, Supporting Information).^[^ ^52^ ^]^ Since the materials in this analysis were usually used as building materials, so one cubic meter of the material was defined as a functional unit.

## Conflict of Interest

The authors declare no conflict of interest.

## Author Contributions

C.X. and H.Y. conceived the project. H.Y., Y.W., and X.J. performed the experiments and analyzed the data. J.W. and L. G. assisted with the EMI SE analysis. J.L., L.C., C.L., and C.X. discussed the results and commented on the manuscript. All the authors discussed the results and commented on the paper.

## Supporting information

Supporting Information

## Data Availability

The data that support the findings of this study are available from the corresponding author upon reasonable request.
