# Supplementary material for: Creation of Wood‐Based Hierarchical Superstructures via In Situ Growth of ZIF‐8 for Enhancing Mechanical Strength and Electromagnetic Shielding Performance
Source: Adv Sci (Weinh). 2024 Feb 21;11(17):2400074. doi: 10.1002/advs.202400074 (PMC11077680; doi:10.1002/advs.202400074)
Supplement: Supplementary file 1 — Supporting Information [file ADVS-11-2400074-s001.pdf]

ADVANCED SCIENCE
Open Access

Supporting Information

for *Adv. Sci.*, DOI 10.1002/advs.202400074

Creation of Wood-Based Hierarchical Superstructures via In Situ Growth of ZIF-8 for Enhancing Mechanical Strength and Electromagnetic Shielding Performance

*Haoran Ye, Ying Wu, Xin Jin, Jiamin Wu, Lu Gan, Jianzhang Li, Liping Cai, Chuangwei Liu and Changlei Xia**

Supplementary Information

Creation of Wood-based Hierarchical Superstructures via In Situ Growth of ZIF-8 for Enhancing Mechanical strength and Electromagnetic Shielding Performance

*Haoran Ye, Ying Wu, Xin Jin, Jiamin Wu, Lu Gan, Jianzhang Li, Liping Cai, Chuangwei Liu and Changlei Xia****Supplementary Figures**

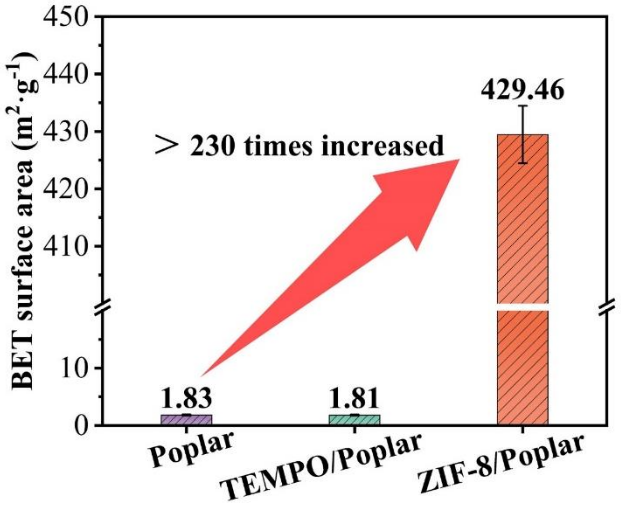

Figure S1. The Brunauer-Emmett-Teller (BET) surface areas of poplar, TEMPO/Poplar composite, and ZIF-8/Poplar composite.

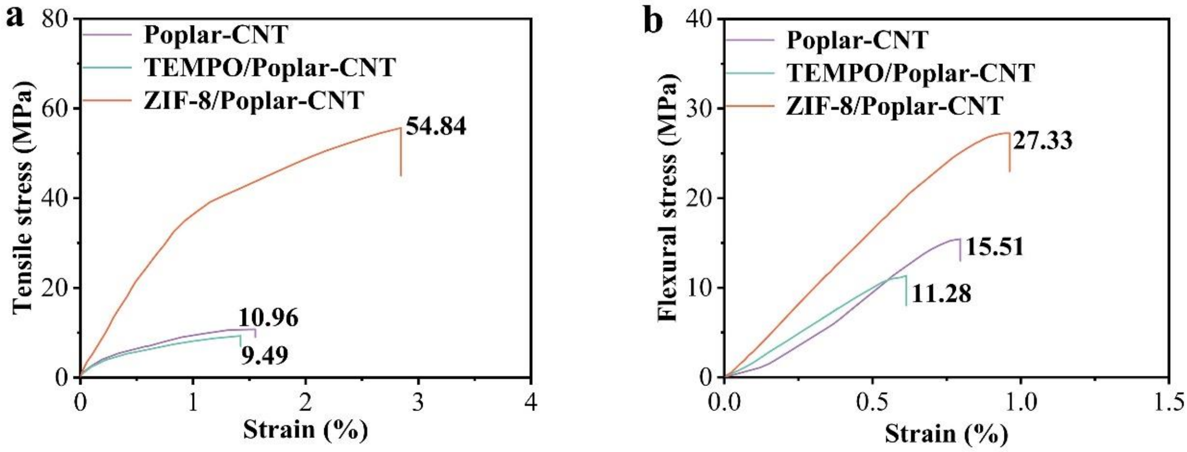

Figure S2. a) Tensile and b) Flexural stress-strain curves of Poplar-CNT composite, TEMPO/Poplar-CNT composite, and ZIF-8/Poplar-CNT composite.

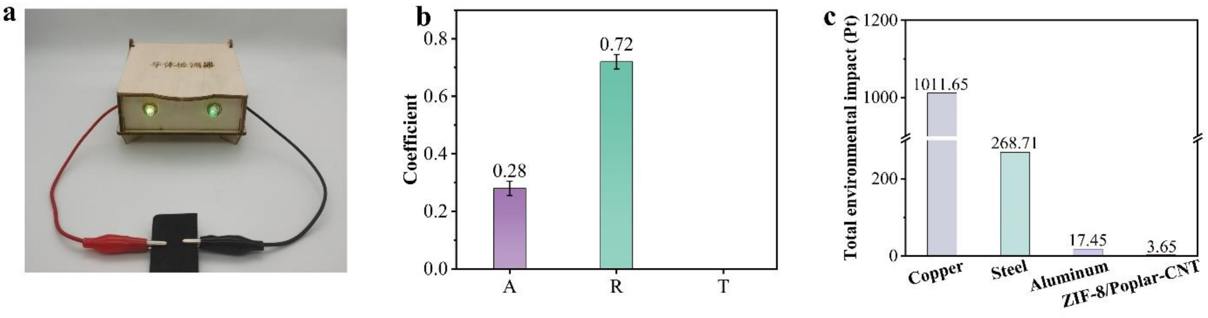

Figure S3. a) Conductivity experiment of ZIF-8/Poplar-CNT composite. b) The average R, A and T values of ZIF-8/Poplar-CNT composite. c) The total environmental impact values of 1 m^3 ZIF-8/Poplar-CNT composite, copper, steel, and aluminium.

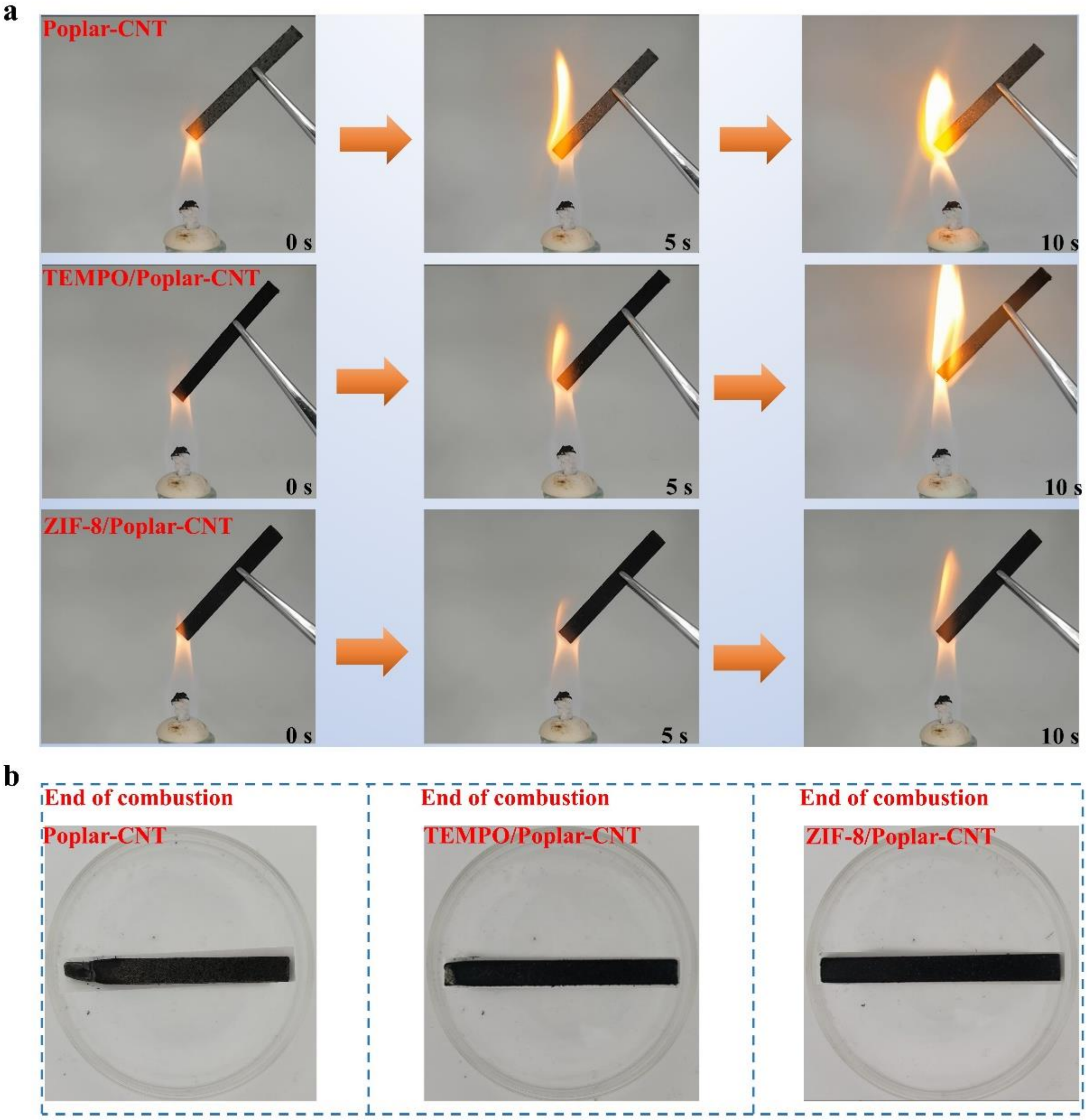

Figure S4. a) The actual burning photographs of Poplar-CNT composite, TEMPO/Poplar-CNT composite, and ZIF-8/Poplar-CNT composite at 0s, 5s, and 10s. b) Poplar-CNT composite, TEMPO/Poplar-CNT composite, and ZIF-8/Poplar-CNT composite at the end of the combustion comparison photographs.

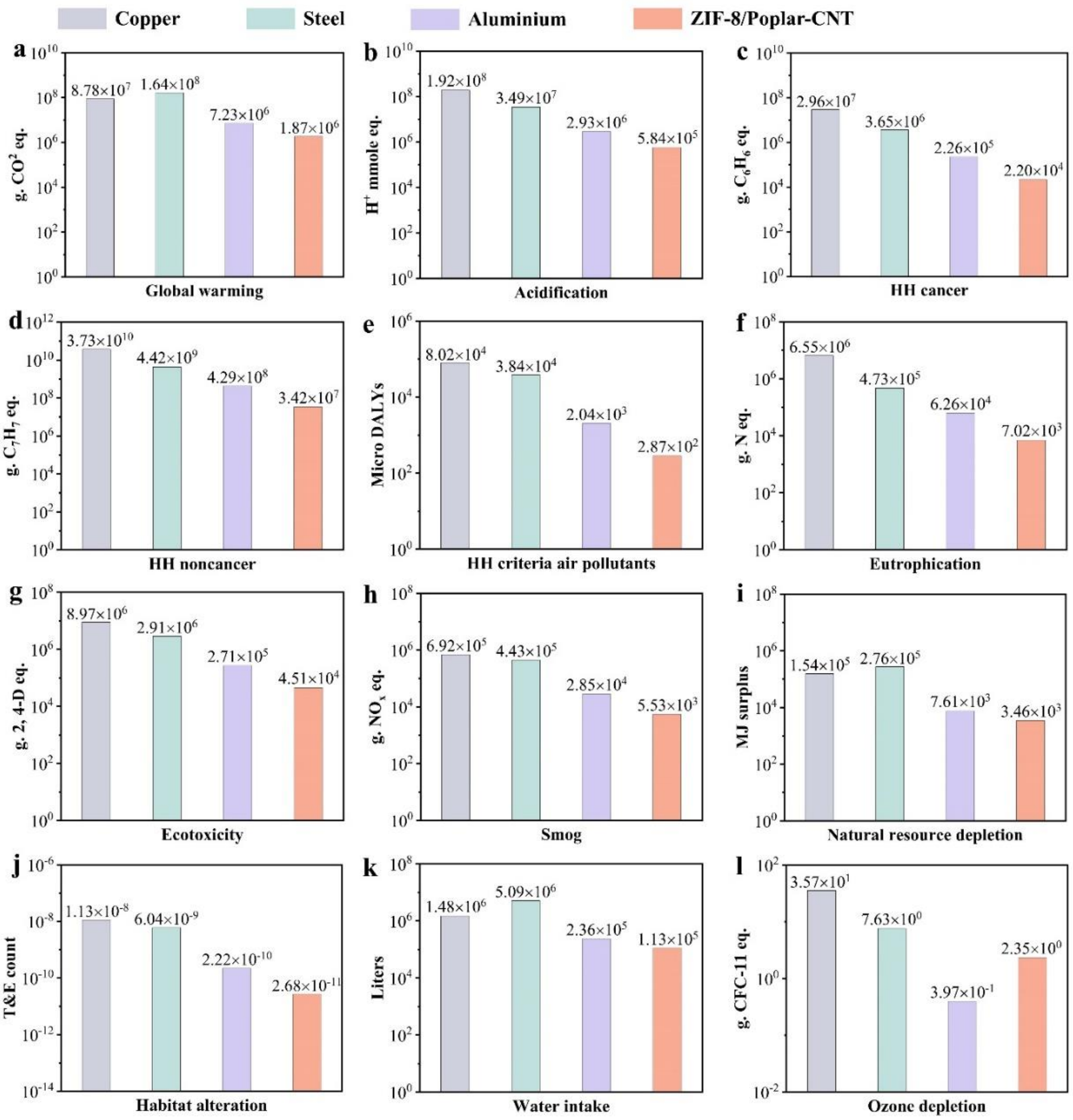

Figure S5. The LCA of 1 m^3 ZIF-8/Poplar-CNT composite, copper, steel, and aluminium, including the (a-h) individual LCI listed in Table S3.

Supplementary Tables

Table S1 Comparison of EMI shielding performance and tensile strength of ZIF-8/Poplar CNT and various typical shielding materials

Materials	Tensile strength (MPa)	EMI SE (dB)	Ref.

MXene/PEDOT:PSS	13.71	42.1	[1]
CNT/Graphene	6.6	47.5	[2]
Ag-NW/PANI	45	51	[3]
RGO/CNF	67	26.2	[4]
MWCNT/PC	36.4	40	[5]
CNT/RN	22.2	44.7	[6]
MWCNT/PU	58.5	29	[7]
PEI/Graphene	3.5	20	[8]
PI/Graphene	11.4	20	[9]
EMA/Graphene	12.5	28	[10]
rGO-$Ti_3C_2T_x$/MXene	24.5	52.6	[11]
$Ti_3C_2T_x$/c-PANI	19.9	36	[12]
PVDF/MXene/AgNW	40	41.26	[13]
ZIF-8/Poplar-CNT	54.84	56.95	This work

Table S2 The sample parameters and SSE/t values of various shielding materials

**Materials**	**EMI SE (dB)**	**Thickness (mm)**	**Density ($g\cdot cm^{-3}$)**	**SSE/t ($dB\cdot cm^2\cdot g^{-1}$)**	**Ref.**
Ni filaments/PES	87	2.85	1.87	165	[14]

PMMA/Graphene	19	2.5	0.79	96.2	[15]
MWCNT/PC	39	2.1	1.13	164	[16]
PEI/Graphene	12.8	2.3	0.29	191.3	[8]
PEI/Graphene/Fe_3O_4	18	2.5	0.41	176	[17]
CB/ABS	20	1.1	0.96	190	[18]
rGO/PEI	12.8	2.3	0.29	176	[8]
SS/PP	48	3.1	0.64	241.9	[19]
rGO/PS	28	2	0.54	257.6	[20]
PMMA-MWCNT/PVDF/ABS	32	3	0.53	201.25	[21]
Graphene/WPU	32	2	1.04	153	[22]
SWCNT/epoxy	25	2	1.73	72	[23]
ZIF-8/Poplar-CNT	56.95	1.91	1.13	263.86	This work

Table S3 A comparison of environmental impact assessment among the five materials

**Impact category (Unit)**	**Copper**	**Steel**	**Aluminum**	**ZIF-8/Poplar-CNT**
Global warming (g. CO_2 eq.)	8.78×10^7	1.64×10^8	7.23×10^6	1.87×10^6

Acidification (H^+ mmole eq.)	1.92×10^{8}	3.49×10^{7}	2.93×10^{6}	5.84×10^{5}
HH cancer (g. C_6H_6 eq.)	2.96×10^{7}	3.65×10^{6}	2.26×10^{5}	2.20×10^{4}
HH noncancer (g. C_7H_7 eq.)	3.73×10^{10}	4.42×10^{9}	4.29×10^{8}	3.42×10^{7}
HH criteria air pollutants (Micro DALYs)	8.02×10^{4}	3.84×10^{4}	2.04×10^{3}	2.87×10^{2}
Eutrophication (g. N eq.)	6.55×10^{6}	4.73×10^{5}	6.26×10^{4}	7.02×10^{3}
Ecotoxicity (g. 2, 4-D eq.)	8.97×10^{6}	2.91×10^{6}	2.71×10^{5}	4.51×10^{4}
Smog (g. NO_x eq.)	6.92×10^{5}	4.43×10^{5}	2.85×10^{4}	5.53×10^{3}
Natural resource depletion (MJ surplus)	1.54×10^{5}	2.76×10^{5}	7.61×10^{3}	3.46×10^{3}
Habitat alteration (T&E count)	1.13×10^{-8}	6.04×10^{-9}	2.22×10^{-10}	2.68×10^{-11}
Water intake (Liters)	1.48×10^{6}	5.09×10^{6}	2.36×10^{5}	1.13×10^{5}
Ozone depletion (g. CFC-11 eq)	3.57×10^{1}	7.63×10^{0}	3.97×10^{-1}	2.35×10^{0}

References

[1] R. Liu, M. Miao, Y. Li, J. Zhang, S. Cao, X. Feng, *ACS Appl. Mater. Interfaces* **2018**, 10, 44787.

[2] Q. Song, F. Ye, X. Yin, W. Li, H. Li, Y. Liu, K. Li, K. Xie, X. Li, Q. Fu, L. Cheng, L. Zhang, B. Wei, *Adv. Mater.* **2017**, 29.

[3] F. Fang, Y.-Q. Li, H.-M. Xiao, N. Hu, S.-Y. Fu, *J. Mater. Chem. C* **2016**, 4, 4193.

[4] W. Yang, Z. Zhao, K. Wu, R. Huang, T. Liu, H. Jiang, F. Chen, Q. Fu, *J. Mater. Chem. C* **2017**, 5, 3748.

[5] S. G. Pardo, L. Arboleda, A. Ares, X. Garcia, S. Dopico, M. J. Abad, *Polym. Compos.* **2013**, 34, 1938.

[6] L.-C. Jia, M.-Z. Li, D.-X. Yan, C.-H. Cui, H.-Y. Wu, Z.-M. Li, *J. Mater. Chem. C* **2017**, 5, 8944.

[7] T. K. Gupta, B. P. Singh, S. R. Dhakate, V. N. Singh, R. B. Mathur, *J. Mater. Chem. A* **2013**, 1, 9138.

[8] J. Ling, W. Zhai, W. Feng, B. Shen, J. Zhang, W. G. Zheng, *ACS Appl. Mater. Interfaces* **2013**, 5, 2677.

[9] Y. Li, X. Pei, B. Shen, W. Zhai, L. Zhang, W. Zheng, *RSC Adv.* **2015**, 5, 24342.

[10] S. Ganguly, S. Ghosh, P. Das, T. K. Das, S. K. Ghosh, N. C. Das, *Polym. Bull.* **2020**, 77, 2923.

[11] Y. Zhang, M.-K. Xu, Z. Wang, T. Zhao, L.-X. Liu, H.-B. Zhang, Z.-Z. Yu, *Nano Res.* **2022**, 15, 4916.

[12] Y. Zhang, L. Wang, J. Zhang, P. Song, Z. Xiao, C. Liang, H. Qiu, J. Kong, J. Gu, *Compos Sci Technol* **2019**, 183.

[13] H. R. Cheng, Y. M. Pan, Q. Chen, R. C. Che, G. Q. Zheng, C. T. Liu, C. Y. Shen, X. H. Liu, *Adv. Compos. Hybrid Mater.* **2021**, 4, 505.

[14] W.-L. Song, X.-T. Guan, L.-Z. Fan, W.-Q. Cao, C.-Y. Wang, Q.-L. Zhao, M.-S. Cao, *J. Mater. Chem. A* **2015**, 3, 2097.

[15] H.-B. Zhang, Q. Yan, W.-G. Zheng, Z. He, Z.-Z. Yu, *ACS Appl. Mater. Interfaces* **2011**, 3, 918.

[16] S. Pande, A. Chaudhary, D. Patel, B. P. Singh, R. B. Mathur, *RSC Adv.* **2014**, 4, 13839.

[17] B. Shen, W. Zhai, M. Tao, J. Ling, W. Zheng, *ACS Appl. Mater. Interfaces* **2013**, 5, 11383.

[18] M. H. Al-Saleh, W. H. Saadeh, U. Sundararaj, *Carbon* **2013**, 60, 146.

[19] A. Ameli, M. Nofar, S. Wang, C. B. Park, *ACS Appl. Mater. Interfaces* **2014**, 6, 11091.

[20] D.-X. Yan, P.-G. Ren, H. Pang, Q. Fu, M.-B. Yang, Z.-M. Li, *J. Mater. Chem.* **2012**, 22, 18772.

[21] G. P. Kar, S. Biswas, S. Bose, *Phys. Chem. Chem. Phys.* **2015**, 17, 14856.

[22] S.-T. Hsiao, C.-C. M. Ma, H.-W. Tien, W.-H. Liao, Y.-S. Wang, S.-M. Li, Y.-C. Huang, *Carbon* **2013**, 60, 57.

[23] Y. Huang, N. Li, Y. Ma, D. Feng, F. Li, X. He, X. Lin, H. Gao, Y. Chen, *Carbon* **2007**, 45, 1614.
